# Histone demethylase JMJD3 regulates CD11a expression through changes in histone H3K27 tri-methylation levels in CD4^+^ T cells of patients with systemic lupus erythematosus

**DOI:** 10.18632/oncotarget.16894

**Published:** 2017-04-06

**Authors:** Heng Yin, Haijing Wu, Ming Zhao, Qing Zhang, Hai Long, Siqi Fu, Qianjin Lu

**Affiliations:** ^1^ Department of Dermatology, Second Xiangya Hospital, Central South University, Hunan Key Laboratory of Medical Epigenomics, Changsha, Hunan, China

**Keywords:** JMJD3, H3K27me3, CD11a, SLE, CD4^+^ T cells

## Abstract

Aberrant CD11a overexpression in CD4^+^ T cells induces T cell auto-reactivity, which is an important factor for systemic lupus erythematosus (SLE) pathogenesis. Although many studies have focused on CD11a epigenetic regulation, little is known about histone methylation. JMJD3, as a histone demethylase, is capable of specifically removing the trimethyl group from the H3K27 lysine residue, triggering target gene activation. Here, we examined the expression and function of JMJD3 in CD4^+^ T cells from SLE patients. Significantly decreased H3K27me3 levels and increased JMJD3 binding were detected within the ITGAL (CD11a) promoter locus in SLE CD4^+^ T cells compared with those in healthy CD4^+^ T cells. Moreover, overexpressing JMJD3 through the transfection of pcDNA3.1-JMJD3 into healthy donor CD4^+^ T cells increased JMJD3 enrichment and decreased H3K27me3 enrichment within the ITGAL (CD11a) promoter and up-regulated CD11a expression, leading to T and B cell hyperactivity. Inhibition of JMJD3 via JMJD3-siRNA in SLE CD4^+^ T cells showed the opposite effects. These results demonstrated that histone demethylase JMJD3 regulates CD11a expression in lupus T cells by affecting the H3K27me3 levels in the ITGAL (CD11a) promoter region, and JMJD3 might thereby serve as a potential therapeutic target for SLE.

## INTRODUCTION

Systemic lupus erythematosus (SLE) is a multifactorial autoimmune connective tissue disease characterized by aberrant lymphocyte auto-reactivity and excess autoantibody production, leading to inflammation and tissue damage in multiple systems. The underlying molecular mechanisms for the autoimmune response remain unclear, although both genetic and epigenetic factors have been implicated [1, 2]. Previous studies have identified more than 60 susceptibility genes associated with lupus, demonstrating the critical role of genetics in lupus autoimmunity [3, 4]. However, the incomplete concordance of lupus in identical twins has not been explained [5, 6]. In recent years, numerous studies have shown that epigenetic alterations triggering T lymphocyte hyperactivation lead to lupus and lupus-like diseases [7–10].

Lymphocyte function-associated antigen 1 (LFA-1) is a member of the integrin family of adhesion proteins and is composed of cluster of differentiation (CD) 11a and CD18 subunits [11]. LFA-1 plays an important role in adhesion between T cells and other immune cells and is also essential to T cell activation, B cell help, leukocyte recruitment, and other processes [12]. LFA-1 has been closely associated with T cell activation and lupus pathogenesis. Adoptive transferring of T cells overexpressing LFA-1 causes lupus-like disease in syngeneic mice [13]. Mice deficient in LFA-1 showed significantly increased survival, decreased anti-DNA autoantibody formation, and reduced glomerulonephritis [14]. LFA-1 overexpression in lupus induces T cell auto-reactivity and B cell autoantibody over-induction [15]. Previous studies have demonstrated that DNA hypomethylation and histone hyperacetylation of the CD11a promoter region contribute to the overexpression of this protein in SLE CD4^+^ T cells [7, 16]. However, the mechanisms responsible for aberrant CD11a overexpression in lupus are not fully understood.

Histone demethylase JMJD3 has received much attention in recent years, due to its specifically regulation of the level of histone H3 lysine 27 tri-methylation (H3K27me3) [17]. H3K27me3 is a hallmark of gene silencing, which plays a role in the transcriptional repression of target genes [18, 19]. Previous studies have confirmed that H3K27me3 enrichment at the CD11a promoter of SLE CD4^+^ T cells is distinctly decreased compared with that of healthy controls and the genomic expression of JMJD3 in CD4^+^ T cells is elevated in SLE patients. Thus, we hypothesized that histone demethylase JMJD3 may participate in regulating CD11a expression in lupus T cells by changing histone H3K27 tri-methylation levels.

In the present study, we investigated the effects of histone demethylase JMJD3 on CD11a in SLE CD4^+^ T cells and examined the underlying regulatory mechanisms. Histone demethylase JMJD3 was overexpressed in the CD4^+^ T cells of SLE patients and increased binding to the CD11a promoter. Further studies have confirmed that JMJD3 promotes CD11a expression by down-regulating H3K27me3 enrichment in the CD11a promoter region, leading to the pathological auto-reaction of SLE. Together, these results provide novel insights into SLE pathogenesis and suggest that JMJD3 may be a novel target for SLE treatment.

## RESULTS

### Enhanced JMJD3 mRNA and protein expression in SLE CD4^+^ T cells

**Figure 1:**
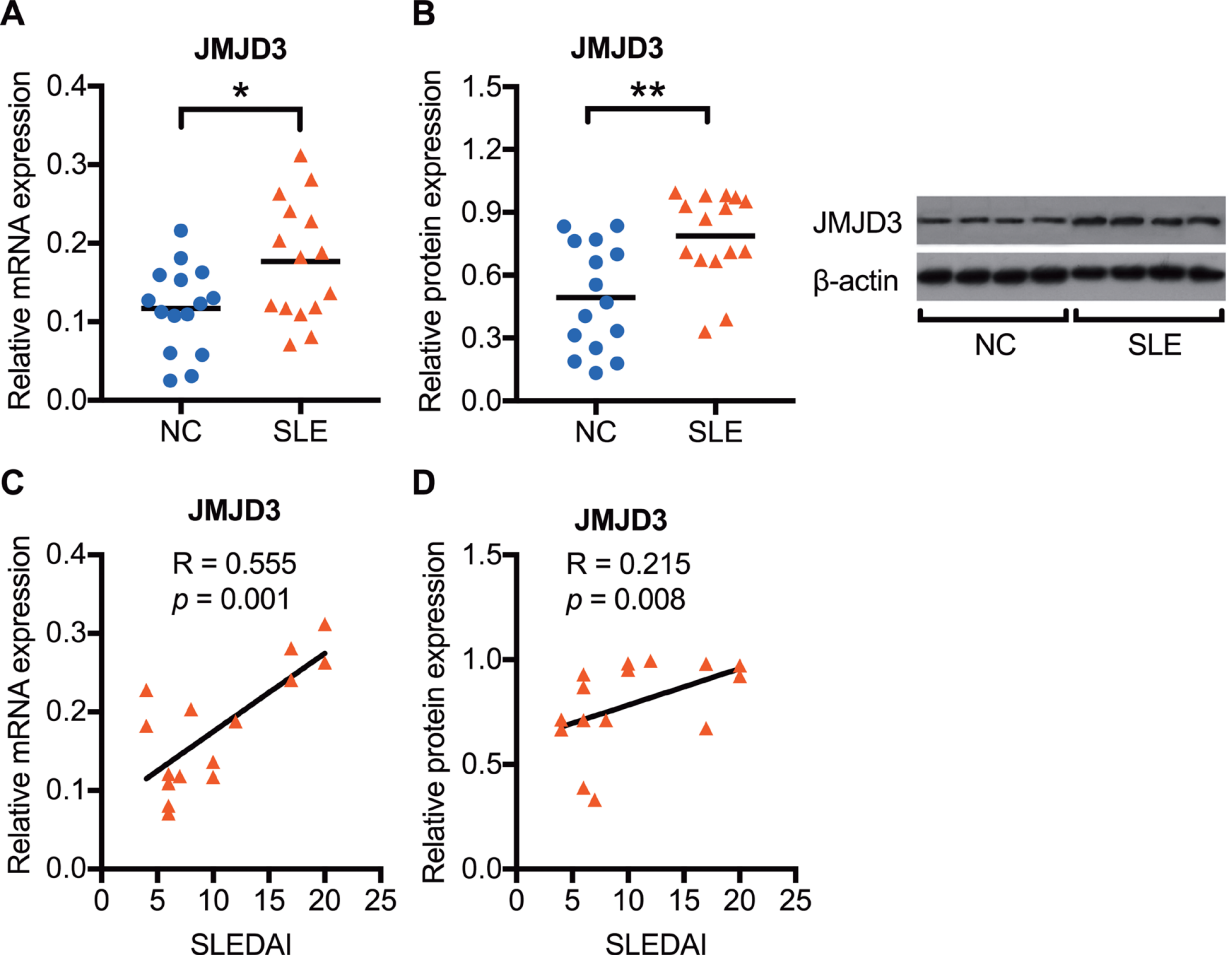
Enhanced JMJD3 mRNA and protein expression in SLE CD4^+^ T cells CD4^+^ T cells were isolated from SLE patients and normal controls. The JMJD3 mRNA and protein expression levels in CD4^+^ T cells were respectively detected using real-time RT-PCR and western blot analysis. (**A**) Relative JMJD3 mRNA expression levels normalized to β-actin in SLE (*n* = 15) and healthy controls (*n* = 15). (**B**) Relative JMJD3 protein expression levels in SLE (*n* = 15) compared with that in healthy controls (*n* = 15). (**C, D**) The correlation analysis of SLEDAI score and JMJD3 expression status in SLE patients. The data are shown by dotplot. *p < 0.05, **p < 0.01, and ***p < 0.001.

To confirm JMJD3 expression changes in SLE, we first detected the mRNA and protein expression levels of JMJD3 using real-time PCR and western blot analysis, respectively. We observed that both the mRNA (Figure 1A) and protein (Figure 1B) expression levels of JMJD3 were significantly enhanced in SLE CD4^+^ T cells (*n* = 15) compared with those of healthy controls (*n* = 15). Furthermore, the SLEDAI score was positively correlated with both mRNA (Figure 1C) and protein (Figure 1D) expression status of JMJD3 in SLE patients.

### Decreased H3K27me3 enrichment and increased JMJD3 binding at the CD11a promoter in SLE CD4^+^ T cells

**Figure 2:**
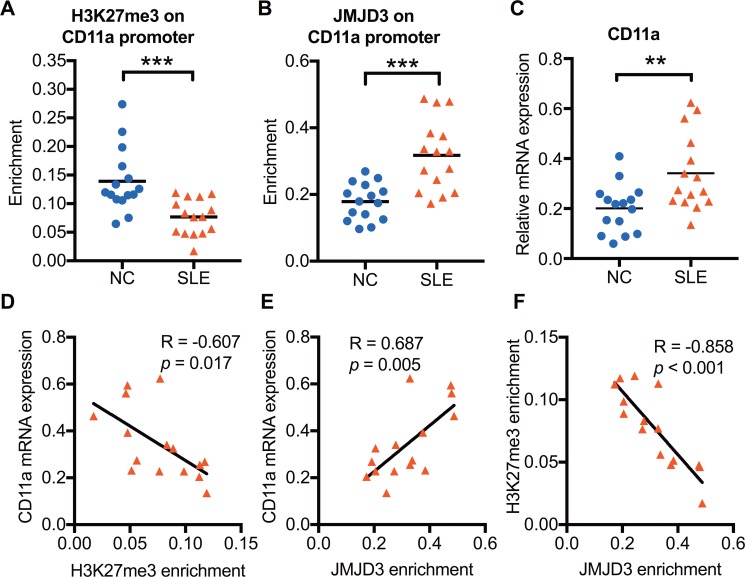
The relationship among H3K27me3 enrichment, JMJD3 enrichment, and CD11a mRNA expression in SLE CD4^+^ T cells CD4^+^ T cells were isolated from SLE patients and normal controls. H3K27me3 enrichment and JMJD3 enrichment within the CD11a promoter in CD4^+^ T cells was assessed using ChIP and real-time PCR. CD11a mRNA expression levels were measured using real-time PCR. (**A, B**) Relative H3K27me3 and JMJD3 enrichment within the CD11a promoter in SLE (*n* = 15) and healthy controls (*n* = 15). The results were normalized to input DNA (total chromatin) and represent the means of three independent experiments. (**C**) Relative CD11a mRNA expression levels in SLE (*n* = 15) and healthy controls (*n* = 15). (**D, E, F**) The correlation analysis of H3K27me3 enrichment, JMJD3 enrichment and CD11a mRNA expression levels in SLE CD4^+^ T cells. The data are shown by dotplot. *p < 0.05, **p < 0.01, and ***p < 0.001.

To verify whether increased JMJD3 affects CD11a expression in SLE CD4^+^ T cells, we further assessed the H3K27me3 enrichment and JMJD3 binding levels within the CD11a promoter of 15 SLE patients and 15 healthy controls using chromatin immunoprecipitation (ChIP) and real-time PCR. Compared with normal controls, a striking decrease of H3K27me3 enrichment (Figure 2A) and a significant increase of JMJD3 binding (Figure 2B) were observed at the CD11a promoter in SLE CD4^+^ T cells. Moreover, we detected the CD11a mRNA expression in CD4^+^ T cells of 15 SLE patients and 15 healthy controls using real-time PCR. Compared with healthy controls, the mRNA expression of CD11a were significantly increased in SLE CD4^+^ T cells (Figure 2C). Interestingly, H3K27me3 enrichment at the CD11a promoter was negatively correlated with CD11a mRNA expression levels in SLE CD4^+^ T cells (Figure 2D). In addition, the levels of JMJD3 binding were positively correlated with CD11a mRNA levels (Figure 2E) but negatively correlated with H3K27me3 enrichment at the CD11a promoter in SLE CD4^+^ T cells (Figure 2F).

### JMJD3 overexpression up-regulates CD11a expression by reducing H3K27me3 enrichment in healthy CD4^+^ T cells

**Figure 3:**
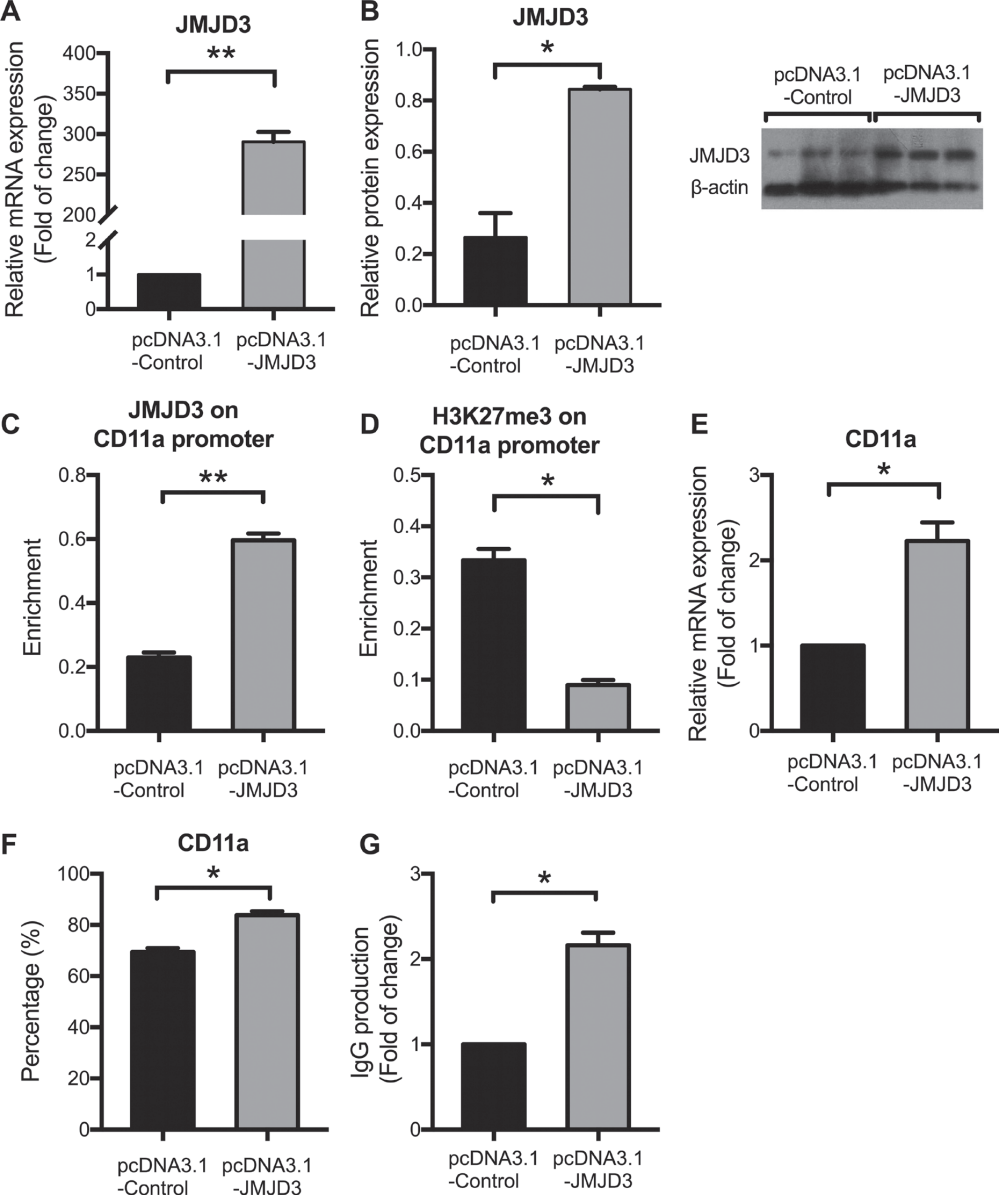
JMJD3 overexpression up-regulates CD11a expression by reducing H3K27me3 enrichment in healthy CD4^+^ T cells CD4^+^ T cells were isolated from healthy controls (*n* = 3) and divided into two groups, which were subsequently transfected with pcDNA3.1-JMJD3 and pcDNA3.1-control, respectively. At 48 h after transfection, relative JMJD3 and CD11a protein levels were assessed using western blot analysis and flow cytometry, respectively. Relative JMJD3 and CD11a mRNA expression levels were measured using real-time PCR. Relative JMJD3 binding and H3K27me3 enrichment at the CD11a promoter in CD4^+^ T cells were assessed using ChIP and real-time PCR. All experiments were performed in triplicate. (**A, B**) Relative JMJD3 mRNA and protein expression levels at 48 h after transfection with pcDNA3.1-JMJD3 and pcDNA3.1-control. β-actin served as an endogenous control. (**C, D**) Relative JMJD3 binding and H3K27me3 enrichment at the CD11a promoter at 48 h after transfection with pcDNA3.1-JMJD3 and pcDNA3.1-control. (**E, F**) Relative CD11a mRNA and protein expression levels at 48 h after transfection with pcDNA3.1-JMJD3 and pcDNA3.1-control. The results of CD11a protein expression are expressed as a percentages (%) of CD4^+^CD11a^+^ T cells. (**G**) Relative IgG production in B cells stimulated by SLE CD4^+^ T cells transfected with pcDNA3.1-JMJD3 or pcDNA3.1-control. The data are shown as the means ± SEM. *p < 0.05, **p < 0.01, and ***p < 0.001.

The increased expression of JMJD3 and decreased enrichment of H3K27me3 within the CD11a promoter suggest that JMJD3 may up-regulate CD11a expression by down-regulating the level of H3K27me3. To confirm this hypothesis, we transfected CD4^+^ T cells from 3 healthy donors with pcDNA3.1-JMJD3 and estimated the consequences on H3K27me3 enrichment and CD11a expression. At 48 h after transfection, JMJD3 expression was significantly enhanced by pcDNA3.1-JMJD3 compared with the pcDNA3.1-control (Figure 3A and 3B). JMJD3 binding to the CD11a promoter was also increased in the pcDNA3.1-JMJD3 group (Figure 3C). Consistently, H3K27me3 enrichment at the CD11a promoter was suppressed after JMJD3 up-regulation (Figure 3D). The CD11a mRNA level (Figure 3E) and the proportion of CD4^+^ T cells expressing CD11a (Figure 3F) were also obviously up-regulated in CD4^+^ T cells transfected with pcDNA3.1-JMJD3 compared with that in the pcDNA3.1-control. To determine whether up-regulating JMJD3 in healthy T cells was sufficient to induce B cell overstimulation, resembling that of lupus T cells, CD4^+^ T cells expressing pcDNA3.1-JMJD3 were cultured with purified autologous B cells (1:4 ratio) for 8 days, and IgG production was then measured using enzyme-linked immunosorbent assays (ELISAs). JMJD3-overexpressing CD4^+^ T cells stimulated IgG synthesis more robustly than control cells (Figure 3G).

### Down-regulating JMJD3 restores aberrant CD11a expression by increasing H3K27 tri-methylation levels in SLE CD4^+^ T cells

**Figure 4:**
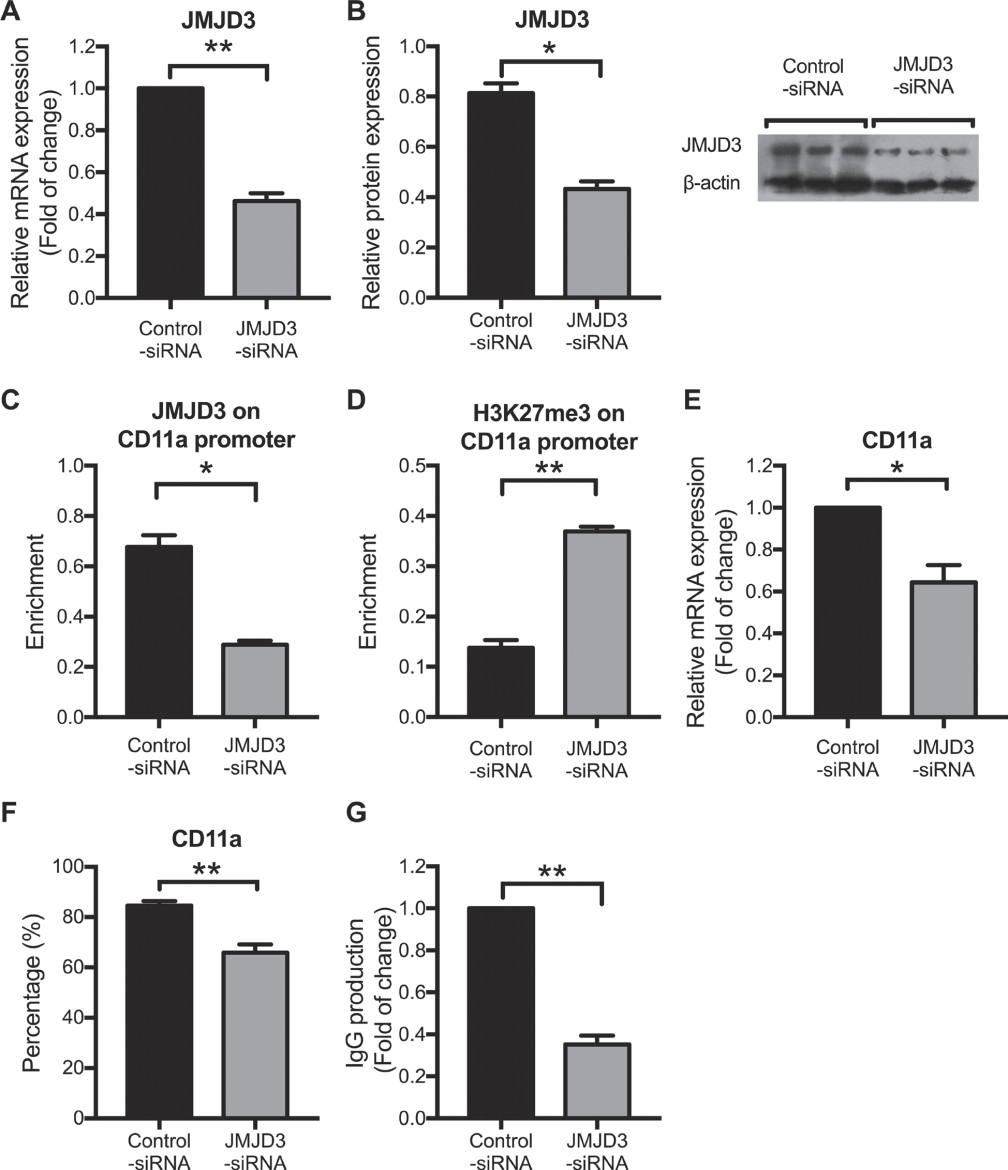
Down-regulating JMJD3 restores aberrant CD11a expression by increasing promoter H3K27me3 levels in SLE CD4^+^ T cells CD4^+^ T cells were isolated from SLE patients (*n* = 3) and divided into two groups, which were subsequently transfected with JMJD3-siRNA and control-siRNA, respectively. At 48 h after transfection, the relative JMJD3 and CD11a protein expression levels were assessed using western blot analysis and flow cytometry, respectively. Relative JMJD3 and CD11a mRNA expression levels were measured using real-time PCR. Relative JMJD3 binding and H3K27me3 enrichment at the CD11a promoter in CD4^+^ T cells were assessed using ChIP and real-time PCR. All experiments were performed in triplicate. (**A, B**) Relative JMJD3 mRNA and protein expression levels at 48 h after transfection with JMJD3-siRNA and control-siRNA. β-actin served as an endogenous control. (**C, D**) Relative JMJD3 binding and H3K27me3 enrichment at the CD11a promoter at 48 h after transfection with JMJD3-siRNA and control-siRNA. (**E, F**) Relative CD11a mRNA and protein expression levels at 48 h after transfection with JMJD3-siRNA and control-siRNA. The results of CD11a protein expression are expressed as percentages (%) of CD4^+^CD11a^+^ T cells. (**G**) Relative IgG production in B cells stimulated by SLE CD4^+^ T cells transfected with JMJD3-siRNA or control-siRNA. The data are shown as the means ± SEM. *p < 0.05, **p < 0.01, and ***p < 0.001.

To verify the effects of JMJD3 on CD11a expression, we also transfected SLE CD4^+^ T cells with a JMJD3-siRNA. At 48 h after transfection, a marked decrease of JMJD3 expression was observed in SLE CD4^+^ T cells compared with that in the siRNA-control cells (Figure 4A and 4B). Concordant decreases in JMJD3 binding to the CD11a promoter were also detected (Figure 4C), while H3K27me3 levels were increased after JMJD3 down-regulation (Figure 4D). Interestingly, CD11a overexpression in SLE CD4^+^ T cells was successfully reversed after siRNA-JMJD3 transfection (Figure 4E and 4F). These results indicate that JMJD3 regulates CD11a expression in SLE CD4^+^ T cells by modulating the status of H3K27me3 at the CD11a promoter. As expected, CD11a down-regulation correlated with a relative decrease in IgG secretion when autologous B cells were co-cultured with JMJD3-suppressing SLE CD4^+^ T cells compared with negative control SLE CD4^+^ T cells (Figure 4G).

## DISCUSSION

In the present study, we reported that SLE was associated with specific changes in the pattern of histone modification within the promoter region of ITGAL, suggesting that aberrant histone methylation status up-regulates CD11a expression in CD4^+^ T cells of SLE patients. First, we confirmed that JMJD3 expression levels were significantly increased in CD4^+^ T cells of SLE patients. Further studies showed increased JMJD3 binding and decreased H3K27me3 enrichment within the CD11a promoter region. After transfecting the JMJD3 expression plasmid into normal CD4^+^ T cells isolated from healthy donors, we observed the up-regulation of JMJD3 binding and the reduction of H3K27me3 enrichment within the CD11a promoter, which increased CD11a expression and enhanced T cell auto-reactivity. In contrast, down-regulating JMJD3 by siRNA-mediated knockdown, reduced JMJD3 binding and increased H3K27me3 enrichment within the CD11a promoter, which suppressed CD11a expression and reversed the auto-reactivity of T cells in SLE patients.

The occurrence of SLE is closely related to the auto-reactivity of T cells, which triggers B cell overactivation and autoantibody overproduction. T cell auto-reactivity has been associated with the overexpression of autoimmune-related genes, and evidence suggests that DNA hypomethylation within the regulatory sequences is involved in the overexpression of autoimmune-related genes, such as CD11a (ITGAL) [16, 20], CD70 (TNFSF7) [8, 16], CD40 ligand (CD40L) [21, 22], perforin (PRF1) [23], and others. Other studies have shown that histone modifications are also involved in the regulation of autoimmune-related genes. Both histone hyperacetylation and lysine 9 demethylation on histone H3 (H3K9) within the promoters of CD11a and CD70 contribute to the genes' overexpression in SLE CD4^+^ T cells [16, 24]. However, despite studies focusing on the role of histone modification in SLE, this mechanism has not been fully understood.

Histone modifications are major regulators of chromatin structure and ultimately effect gene transcription by controlling the level of chromatin condensation. H3K27me3 is one of the key post-translational modifications of histones, which can be catalyzed to mono-, di-, or tri-methylated states. The mono-methylation of H3K27 (H3K27me) is associated with gene activation, whereas H3K27me3 is associated with gene repression [25]. H3K27me3 suppresses gene expression primarily by recruiting or stabilizing the polycomb repressive complex 1 (PRC1) on chromatin and subsequently blocking the access of transcriptional activation factors and chromatin remodeling factors to DNA [26, 27]. The results of the present study indicated that H3K27me3 levels within the CD11a promoter region of SLE CD4^+^ T cells were significantly decreased and negatively correlated with CD11a mRNA expression. Therefore, we speculated that H3K27me3 might be a negative regulatory factor involved in the regulation of CD11a expression in CD4^+^ T cells of SLE.

H3K27me3 enrichment can be regulated in a biphasic manner primarily catalyzed by the histone demethylases JMJD3 and UTX [17, 28–30] and the histone methylase EZH2 [31, 32]. Studies on JMJD3 have attracted much attention in recent years, as this protein is the specific demethylase of H3K27me3 [17]. The increased binding of JMJD3 within regulatory sequences mediates the specific demethylation of H3K27me3, associated with gene activation, whereas the decreased binding of JMJD3 results in H3K27me3 elevation and inhibits gene transcription. In the present study, we observed a significant increase in JMJD3 binding to the CD11a promoter region, which was positively correlated with CD11a mRNA expression and negatively correlated with H3K27me3 enrichment in the same region. Further studies have shown that up-regulating JMJD3 in healthy donor CD4^+^ T cells increased JMJD3 binding and decreased H3K27me3 enrichment within the ITGAL (CD11a) promoter, thereby causing T and B cell hyperactivity, while inhibiting JMJD3 in SLE CD4^+^ T cells showed the opposite effects. Therefore, we proposed that JMJD3 regulates CD11a expression by changing histone H3K27me3 enrichment in CD4^+^ T cells of SLE patients. Since these manipulations not only affected JMJD3 promoter binding but also altered total JMJD3 levels, at this time, we cannot eliminate the likelihood that JMJD3 also regulates CD11a in other ways.

In conclusion, the results of the present study indicated that JMJD3 binding within the CD11a promoter region was increased in SLE CD4^+^ T cells, while H3K27me3 was reduced in the same area. These two factors promote CD11a expression, leading to abnormal T cell reactivity and ultimately contributing to SLE-mediated autoimmunity. These findings also revealed that JMJD3 was subjected to epigenetic regulation in SLE CD4^+^ T cells, suggesting that JMJD3 might serve as an important target for effective SLE therapy.

## MATERIALS AND METHODS

### Patients and controls

A total of 18 SLE patients (mean age, 29.06±2.03 years) were recruited from the Department of Dermatology, Second Xiangya Hospital, Central South University. All patients fulfilled at least four of the SLE classification criteria of the American College of Rheumatology [33], and the disease activity was assessed using the SLE Disease Activity Index (SLEDAI)[34]. The demographics and medication information for the SLE patients are shown in Table 1. The clinical data and autoantibody profile of SLE patients were shown in Supplementary Tables 1 and 2 (see Supplementary Material), respectively. A total of 18 healthy controls (mean age, 28.06 ± 1.62 years) were recruited from the staff at Second Xiangya Hospital. Written informed consent was obtained from all patients and normal controls who were age- and sex-matched in all experiments. The present study was approved by the Human Ethics Committee of the Central South University Second Xiangya Hospital.

**Table 1:** Patient demographics and medications

Patient	Gender	Age (years)	SLEDAI	Medications
1	Female	47	17	Pred 50 mg/d, TGP 1.8 g/d, HCQ 0.2 g/d
2	Female	15	12	None
3	Female	24	6	Pred 40 mg/d, LEF 10 mg/d, HCQ 0.4 g/d
4	Female	25	6	Pred 12.5 mg/d, TGP 1.8 g/d, HCQ 0.2 g/d
5	Female	22	10	Pred 20 mg/d, MMF 1.0 g/d, HCQ 0.2 g/d
6	Female	33	6	Pred 10 mg/d
7	Female	36	10	Pred 50 mg/d
8	Female	31	7	Pred 20 mg/d, HCQ 0.2 g/d
9	Female	27	6	Pred 40 mg/d
10	Female	21	17	Pred 40 mg/d
11	Male	23	20	MMF 1.5 g/d, HCQ 0.2 g/d
12	Female	33	8	Pred 5 mg/d, TGP 1.8 g/d
13	Female	22	20	None
14	Female	27	4	Pred 10 mg/d
15	Male	32	4	Pred 15 mg/d
16^a^	Female	28	10	Pred 10 mg/d
17^a^	Female	49	5	None
18^a^	Female	28	2	Pred 50 mg/d, HCQ 0.2 g/d

Abbreviations: Pred, Prednisone; HCQ, Hydroxychloroquine; TGP, Total Glucosides of Paeony; MMF, Mycophenolate Mofetil; LEF, Leflunomide.

^a^These 3 SLE samples were used for transfection.

### T cell isolation and culture

A total of 60 ml of venous peripheral blood was withdrawn from each SLE patient and healthy control and preserved with heparin. Peripheral blood mononuclear cells (PBMCs) were isolated using Ficoll-Hypaque density gradient centrifugation (GE Healthcare, Chicago, Illinois, United States). CD4^+^ T cells were isolated by positive selection using magnetic beads, according to the manufacturer's instructions (Miltenyi Biotec, Bergisch Gladbach, Germany). The purity of enriched T cells was generally higher than 95%, as evaluated using flow cytometric analysis. The isolated CD4^+^ T cells were subsequently cultured in human T cell culture medium (Lonza, Walkersville, MD, USA).

### T cell transfection

Both pcDNA3.1-JMJD3-expressing plasmid and pcDNA3.1 blank plasmid were kindly provided from Dr. Charlie Degui Chen (Chinese Academy of Sciences). JMJD3-siRNA and control-siRNA were synthesized at RiboBio (Guangzhou, China). CD4^+^ T cells were transfected with plasmids or siRNAs using the Human T cell Nucleofector Kit and Amaxa Nucleofector, according to manufacturer's instructions (Lonza, Walkersville, MD, USA). The transfected cells were rested in human T cell culture medium containing 10% fetal bovine serum (FBS) for 48 h prior to further analysis.

### RNA isolation and real-time quantitative RT-PCR

Total RNA was isolated from CD4^+^ T cells using an RNeasy Mini Kit (Qiagen, Valencia, CA, USA) and stored at −80°C. Real-time quantitative RT-PCR was performed using a Rotor-Gene 3000 (Corbett Research, Mortlake, NSW, Australia). The mRNA levels were quantified using the One-Step SYBR PrimeScript RT-PCR Kit (TaKaRa Biotechnology [Dalian] Co., Ltd. China). β-actin was selected as the endogenous control, and water was used instead of RNA as the negative control. The following primer pairs were used: for JMJD3, forward 5′ -TTCTCTCCGTCAACATCAA-3′ and reverse 5′ -AGGAACCCGTCAAGTAGTCC-3′; for CD11a, forward 5′-AAATGGAAGGACCCTGATGCTC-3′ and reverse 5′-TGTAGCGGATGATGTCTTTGGC-3′; for β-actin, forward 5′-CGCGAGAAGATGACCCAGAT-3′ and reverse 5′-GCACTGTGTTGGCGTACAGG-3′. The PCR analysis was conducted in triplicate for each sample.

### Western blot

Western blots were performed as previously described [35]. The following primary antibodies were used: anti-JMJD3 (1:875; Abcam, MA, USA) and anti-β-actin (1:2000; Santa Cruz, CA, USA). The blots were visualized using SuperSignal West Pico Chemiluminescent Substrate (Pierce, Rockford, IL, USA) and exposed to X-ray film. The experiments were repeated three times, and the relative expression levels were quantified using Quantity One software (Bio-Rad, Hercules, CA, USA).

### Flow cytometric analysis

At 48 hours after transfection, the CD4^+^ T cell suspension (100 μl; 1 × 10^5^ cells) was incubated with fluorescein isothiocyanate (FITC)-conjugated anti-human CD11a (Becton Dickinson, San Jose, CA, USA) for 20 min at room temperature, washed in 2 ml of Stain Buffer (pH 7.4) containing 0.2% (w/v) bovine serum albumin (BSA) (Becton Dickinson, San Jose, CA, USA), and centrifuged at 400 x g for 5 min. The pelleted cells were subsequently resuspended in 0.5 ml of PBS/BSA. The FACSCalibur flow cytometry system (Becton Dickinson, Franklin Lakes, NJ, USA) was used to acquire fluorescence signal, and the results were subsequently analyzed using CellQuest software (Becton Dickinson, Franklin Lakes, NJ, USA). Relative CD11a protein expression is shown as the MFI and the percentage of cells expressing CD11a.

### T and B cell costimulation assays

B cells were isolated by positive selection using CD19 magnetic beads (Miltenyi Biotec, Bergisch Gladbach, Germany) and cultured in RPMI 1640 medium supplemented with 10% FBS, 100 U/ml penicillin G, and 100 μg/ml streptomycin. At 48 h post-transfection, CD4^+^ T cells (1 × 10^5^ cells) were co-cultivated with autologous B cells (4 × 10^5^ cells) at a ratio of 1:4 as previously described [8]. The cells were cultured in 24-well round-bottomed plates (Costar, Corning NY, USA) containing a total volume of 250 μl for 8 days and supplemented with 250 μl of medium on day 4. The supernatants were collected on day 8 to measure the IgG concentration.

### IgG ELISAs

The total IgG concentration in the supernatants of cells harvested from T-B cell co-cultures were determined using an IgG quantification ELISA kit (Columbia Bio LLC, Elmhurst, USA), according to the manufacturer's instructions. Three replicate wells were used for every sample, and all experiments were performed in triplicate. The absorbance of the samples at 405 nm was measured using an ELx800 Absorbance Microplate Reader (BioTek, Winooski, VT, USA).

### Chromatin immunoprecipitation (ChIP)

ChIP assays were performed using a ChIP Assay Kit (Millipore, Billerica, MA, USA) according to the manufacturer's instructions. Briefly, CD4^+^ T cells were crosslinked with 1% formaldehyde and sonicated to shear the DNA to 500 ∼ 1,000-bp fragments. Protein/DNA complexes were precipitated with histone-specific antibodies. The H3K27me3 and JMJD3 antibodies were purchased from Abcam (MA, USA). Protein-DNA crosslinks were reversed during incubation at 65°C, and precipitated DNA was then extracted with phenol / chloroform and further purified using ethanol prior to the amplification of the target DNA by real-time quantitative PCR (RT-qPCR). The following primer pairs were used: CD11a, forward 5′-AAATGGAAGGACCCTGATGCTC-3′ and reverse 5′-TGTAGCGGATGATGTCTTTGGC-3′.

### Statistical analysis

GraphPad Prism software (version 7.0a for Mac OS X, La Jolla California USA, www.graphpad.com) was used for statistical analysis. The data are reported as the means ± SEM. The data were first analyzed using the Kolmogorov-Smirnov test and one-way ANOVA. Subsequently, the independent two-sample *t-test* was used to compare the means of two groups with Gaussian distribution, whereas the Mann-Whitney test was used to compare the means of two groups with skewed distribution. The paired-samples *t-test* was used to examine paired samples with a normal distribution of differences. Correlations were determined using Pearson's correlation coefficient. *P* values less than 0.05 were considered significant.

## SUPPLEMENTARY TABLES



## Abbreviations

SLE: Systemic lupus erythematosus
LFA-1: Lymphocyte function-associated antigen 1
CD: cluster of differentiation
H3K27me3: histone H3 lysine 27 tri-methylation
SLEDAI: SLE Disease Activity Index
Pred: Prednisone
HCQ: Hydroxychloroquine
TGP: Total Glucosides of Paeony
MMF: Mycophenolate Mofetil
LEF: Leflunomide
PBMCs: Peripheral blood mononuclear cells
FBS: fetal bovine serum
FITC: fluorescein isothiocyanate
BSA: bovine serum albumin
MFI: mean fluorescence intensity
ELISAs: IgG enzyme-linked immunosorbent assays
ChIP: Chromatin immunoprecipitation
WB: Western blot
RT-qPCR: real-time quantitative PCR
H3K27me: Mono-methylation of H3K27
PRC1: polycomb repressive complex 1
