# Supplementary material for: Histone demethylase JMJD3 regulates CD11a expression through changes in histone H3K27 tri-methylation levels in CD4+ T cells of patients with systemic lupus erythematosus
Source: Oncotarget. 2017 Apr 6;8(30):48938–47. doi: 10.18632/oncotarget.16894 (PMC5564738; doi:10.18632/oncotarget.16894)
Supplement: Supplementary file 1 [file oncotarget-08-48938-s001.pdf]

Histone demethylase JMJD3 regulates CD11a expression through changes in histone H3K27 tri-methylation levels in $CD4^+$ T cells of patients with systemic lupus erythematosus

Supplementary Materials

Supplementary Table 1: Clinical data of patients

Patient	Clinical manifestations	Laboratory test	SLEDAI score
1	Vasculitis, New rash, Mucosal ulcers	Hematuria, Leukopenia	17
2	Arthritis, New rash, Alopecia, Mucosal ulcers, Fever	Leukopenia	12
3	New rash, Alopecia	Low complement	6
4	Alopecia	Low complement, Increased DNA binding	6
5	None	Hematuria, Proteinuria, Low complement	10
6	None	Pyuria, Low complement	6
7	Arthritis, New rash, Alopecia	Low complement	10
8	Fever	Proteinuria, Low complement	7
9	New rash, Mucosal ulcers	Increased DNA binding	6
10	Arthritis, New rash	Proteinuria, Pyuria, Increased DNA binding, Leukopenia	17
11	New rash, Mucosal ulcers	Urinary casts, Hematuria, Proteinuria, Pyuria	20
12	Visual disturbance	None	8
13	Arthritis, New rash, Alopecia, Mucosal ulcers	Hematuria, Proteinuria, Low complement	20
14	Alopecia	Low complement	4
15	Mucosal ulcers	Low complement	4
16	None	Hematuria, Proteinuria, Low complement	10
17	New rash, Fever	Low complement	5
18	None	Low complement	2

Supplementary Table 2: Autoantibody profile of patients

Patient	1	2	3	4	5	6	7	8	9	10	11	12	13	14	15	16	17	18
ANA	1:40N	1:40S	1:80S	1:20S	1:20S	1:80S	1:80H	1:160S	1:40S	1:40S	1:40S	1:80S	1:80S	1:40S	1:80N	1:80S	1:20N	1:40S
U1-nRNP	-	-	+++	++	+	+++	-	-	-	-	+++	-	+++	+++	-	+++	-	++
Sm	-	-	-	-	-	+++	-	-	-	-	+	-	-	+	-	+++	-	-
SS-A	++	+++	++	-	-	+++	+++	+	++	-	+++	-	-	+++	+++	-	-	+++
Ro-52	-	+++	-	-	-	+++	+++	-	++	-	+++	-	-	+++	+++	-	-	+
SS-B	+++	++	-	-	-	-	-	+	+++	-	+++	-	-	-	-	-	-	-
Scl-70	-	-	-	-	-	-	-	-	-	-	-	-	-	-	-	-	-	-
PM-Scl	-	-	-	-	-	-	-	-	-	-	-	-	-	-	-	-	-	-
Jo-1	-	-	-	-	-	-	-	-	-	-	-	-	-	-	-	-	-	-
Centromere B	-	-	-	-	-	-	-	-	-	-	-	-	-	-	-	-	-	-
PCNA	-	-	-	+	-	-	-	-	-	-	-	-	-	-	-	-	-	-
dsDNA	-	-	-	+	-	-	-	-	++	++	-	-	-	-	-	-	-	-
Nucleosomes	-	-	-	+	-	-	+	-	-	++	-	-	-	-	++	-	-	-
Histones	-	-	-	+	-	-	++	-	+++	+++	-	-	-	-	-	-	-	-
Ribosomal-P Protein	-	+++	-	++	-	+	++	-	-	-	-	-	-	+	+++	+++	-	-
AMA-M2	-	-	-	-	-	-	-	-	-	+	-	-	-	-	-	-	-	-

H, Homogeneous; N, Nucleolar; S, Speckle.
